# Treatment of uveitis: Beyond steroids

**DOI:** 10.4103/0301-4738.58466

**Published:** 2010

**Authors:** Virender S Sangwan

**Affiliations:** Associate Director L V Prasad Eye Institute, Head - Cornea and Anterior Segment, Ocular Immunology and Uveitis Service, L.V. Prasad Eye Institute, Kallam Anji Reddy Campus, L.V. Prasad Marg, Banjara Hills, Hyderabad - 500 034, India. E-mail: vsangwan@lvpei.org

Intraocular inflammatory disease, or uveitis, is a serious problem for the ophthalmic practitioner. It affects patients in the working age group, and is a Th-1 mediated autoimmune disease. The average annual incidence[1] of uveitis has been reported as approximately 14-17 per 100,000, with a peak in the 20-50 years age group, and then it declines. The total population prevalence of uveitis varies geographically; 38 per 100,000 in France, around 200 per 100,000 in the United States, and is estimated to be 730 per 100,000 in India.[2] For India this translates into 8.5 million people with uveitis for an estimated population of 1168 million in 2010. It can cause devastating visual loss and is the fifth commonest cause of visual loss in the developed world, accounting for about 10-15% of the cases of total blindness and up to 20% of legal blindness.[1] In most cases, vision loss arises from cumulative damage to ocular tissues that results from recurrent or chronic inflammation rather than from an isolated attack of an acute inflammatory episode. Therefore, control of inflammation in noninfectious uveitis is critical to minimize the vision loss.

More than 50 years ago, the treatment of uveitis underwent a tremendous paradigm shift with the introduction of corticosteroids into the ophthalmic therapeutic armamentarium. Although corticosteroids are initially effective in many patients, the adverse effects associated with their continued administration make them unacceptable as a long-term systemic therapy. The recent use of intraocularly placed steroids is still being investigated, and has limitations due to local adverse effects, treatment in bilateral cases, and sustained long-term therapy in chronic/recurrent cases. The need for less toxic, effective anti-inflammatory treatment inspired the use of immunosuppressive drugs for ocular inflammatory disease, beginning in the late 1970s. The approach is based on similar principles and agents to those that have been applied in the rheumatology and organ transplantation settings for many years. Knowledge about the potential side-effects of these agents has been well-established due to its use in these fields.

Use of immunosuppressive therapy for inflammatory eye disease has been advocated by an expert panel[3] in three settings: as corticosteroid-sparing therapy when the disease can be controlled with oral corticosteroids but expected toxicity is high at the dose required; for inflammation recalcitrant to oral corticosteroids; and for management of specific diseases, such as Wegener's granulomatosis, sympathetic ophthalmia, and Vogt-Koyanagi Harada's disease, expected to fare poorly with lower levels of therapy. The immunosuppressive agents most commonly used for the treatment of ocular inflammatory disease include the antimetabolites azathioprine, methotrexate, and mycophenolate mofetil (MMF); the T-cell inhibitors cyclosporine and tacrolimus; and alkylating agents chlorambucil and cyclophosphamide. The patients must be adequately immunosuppressed yet be spared the potentially serious consequences of drug toxicity. In the hands of a physician trained in their use and monitoring, the administration of immunosuppressive agents appear to produce fewer side-effects than chronic use of systemic steroids. The safe use of these drugs begins with exclusion of infectious, mechanical, or other treatable causes of ocular inflammation. Diagnostic studies are then obtained, both based on careful review of the system and from physical findings. Where possible, biopsy and histological examination of inflamed tissue is performed (e.g., conjunctival biopsy in patients with ocular cicatricial pemphigoid), because they provide the most reliable guide to the nature of an underlying immunopathologic process. The choice of the immunosuppressive agents is individualized for each patient and depends on a variety of considerations, including the underlying disease, the patient's age, sex, and medical status. Patients are carefully screened for risk factors that might preclude the use of certain immunosuppressive agents (i.e., hepatic disease for methotrexate and renal disease for cyclosporine). Patients are also informed of the proper dosage and regimen, potential adverse reactions, and alternatives to immunosuppressive therapy. The responsibility of the details of the management of patients requiring immunosuppressive therapy must lie with the clinician, who, by virtue of training and experience, is truly expert in the use of these agents and in the recognition and treatment of potentially serious side-effects that may arise. A “hand-in-glove” collaboration between the ophthalmologist and the chemotherapist – usually, in our experience, an oncologist or hematologist--works most effectively for patients requiring such medications.

Periodic complete hemograms, including differential and platelet values, should be obtained in all patients before therapy is initiated and again at one to four weeks' interval to monitor for myelosuppression. We should avoid depressing the leukocyte count below 3500 cells/ul or neutrophil count below 1500 cells/ul and avoid thrombocytopenia less than 75,000 platelets/ul. In addition, liver function tests, urinalysis, blood urea nitrogen (BUN), and serum creatinine should be obtained before initiation of therapy and at intervals of one to four months, depending on the medication. If an adequate clinical response is not observed after minimum of three months of treatment at the maximal tolerable dosage or if toxicity precludes continuation of therapy, the medication should be discontinued and consideration be given to substituting an alternate immunosuppressive agent. If, instead, a good clinical response is obtained and the patient is free of cellular inflammatory activity in the eye, the drug may be tapered and discontinued in most patients after two years of therapy if their disease does not recur. The safety and efficacy of immunosuppressive therapy is well established in ophthalmic literature.[4]

Understanding the mechanisms that lead to uveitis has been aided by the study of inflammatory disease models, particularly experimental autoimmune uveoretinitis (EAU). The clinical and pharmacologic features of EAU are very similar to those of clinical human uveitis, except that the laboratory disease is not spontaneous but induced. Using this model, which can be induced with a variety of uveitogenic antigens found in the posterior segment of the eye, has resulted in a dissection of the major components of the inflammatory response. This has resulted in the development of more specific, effective, and safer therapeutic agents. That information has brought us into a new era of therapy for these disorders, which will soon change the paradigm of the therapeutic approach.

## Biologics in the treatment of Uveitis

Pro-inflammatory chemokines such as tumor necrosis factor alpha (TNF-alpha), interleukins 1,2 and 6 and interferon gamma (IFN-gamma) play a key role in the pathogenesis of non-infectious uveitis. It is against these chemokines and their respective receptors that some biologic agents are designed to act, whilst other biologic agents are designed to counteract the secretors of these chemokines, T- and B-cells, thereby aiming to prevent a downward cascade of inflammation. These agents are not only antibodies and antagonists but are also small molecules that inhibit cellular interactions that modulate inflammatory response. These agents are also termed as “biologic response modifiers”. The use of anti-TNF-alpha agents has revolutionized the treatment of chronic refractory inflammatory disorders.[6] Its efficacy has been proven beyond doubt in the treatment of systemic diseases such as rheumatoid arthritis (RA), juvenile idiopathic arthritis (JIA), as well as endogenous, non-infectious refractory uveitis associated with Behcet's disease and sarcoidosis. Of the several anti-TNF-alpha agents available, three agents have been described in ocular inflammatory conditions- infliximab, adalimumab, and etanercept. Infliximab appears to be the most promising and most extensively studied.[6] Since all these agents affect normal immune response they compromise protection against infections. Tuberculosis must be excluded before starting treatment with biologics as there is a fivefold increased risk of activation.

## Next-generation calcineurin inhibitors (CNIs) for treatment of uveitis

Calcineurin inhibitors (CNIs) are potent immunosuppressants that reversibly inhibit T-cell proliferation and prevent the release of pro-inflammatory cytokines by blocking the activity of calcineurin, a ubiquitous enzyme that is found in cell cytoplasm. CNIs can be highly effective in immune-mediated uveitis. Voclosporin is a CNI which has been shown to be effective in controlling uveitis in the EAU model by inhibiting lymphocyte proliferation.[5] This, a rationally designed novel CNI, exhibits a favorable safety profile, strong correlation between pharmacokinetics and pharmacodynamic response, and a wide therapeutic window. The LUMINATE (Lux Uveitis Multicenter Investigation of a New Approach to Treatment) clinical development program[6] was initiated in 2007 to assess the safety and efficacy of voclosporin for treatment, maintenance, and control of all forms of noninfectious uveitis. Two hundred and eighteen patients were enrolled in this study at 57 centers in America, Europe, and India. The target dose of 0.4 mg/kg twice daily was statistically superior to placebo at both 16- and 24 weeks in controlling inflammation.[7]

In summary, corticosteroids may still be the mainstay of therapy in an acute attack of uveitis but there are several safe and effective alternative therapeutic options available to the clinician for long-term management of uveitis. Locally sustained release device for steroids is another new development and specific immunomodulatory agents are the future for better management of ocular inflammatory diseases.
